# Marjolin’s ulcer in an ischial pressure sore presented with necrotizing soft tissue infection: A case report

**DOI:** 10.1097/MD.0000000000033450

**Published:** 2023-03-31

**Authors:** Ching-Yen Huang, Zhoa-Yu Hsieh, Ke-Chung Chang, Dun-Hao Chang

**Affiliations:** a Division of Plastic and Reconstructive Surgery, Department of Surgery, Far Eastern Memorial Hosptial, New Taipei, Taiwan; b Division of Medical Imaging, Department of Radiology, Far Eastern Memorial Hospital, New Taipei, Taiwan; c School of Medicine, National Yang Ming Chiao Tung University, Hsinchu, Taiwan.

**Keywords:** infection, Marjolin’s ulcer, pressure ulcer, skin cancer

## Abstract

Marjolin’s ulcer (MU) is a rare skin malignancy derived from a chronic wound. Pressure ulcer related MU possesses poor prognosis and high metastatic rate, and it is difficult to be differentiated, especially when superimposed infection.

**Patient concerns:**

Here we report a case with pressure ulcer related MU which presented as necrotizing soft tissue infection (NSTI) to demonstrate the manifestation, treatment, and prognosis of this rare disease.

**Diagnoses:**

A 45-year-old male patient had spinal cord injury at age 2 years. He presented ischial pressure sore complicated with NSTI initially. After serial debridements and antibiotic treatment, the infection subsided. For the persistent verruca-like skin lesion, he underwent wide excision which revealed well-differentiated squamous cell carcinoma. Further image studies showed localized residual tumor without distant metastasis.

**Interventions:**

He then underwent hip disarticulation and anterior thigh fillet flap reconstruction. Local recurrence developed 3 months later, and re-wide excision and inguinal lymph node dissection were performed. No lymph node metastasis was noted and adjuvant radiotherapy was given.

**Outcomes:**

He was followed for 34 months and no recurrence or metastasis was found. The patient can move with a wheelchair or a hip prosthesis, and is partially dependent for daily activities.

**Lessons:**

MU can masquerade as NSTI and one should be alert to its malignant potential. Due to its aggressive nature, limb sacrifice can be considered in circumstances of profound involvement. As for the reconstruction method, pedicled fillet flap provided good wound coverage.

## 1. Introduction

Marjolin’s ulcer (MU) is a rare form of skin malignancy that arises from damaged skin such as burns, trauma, venous stasis ulcers, or other chronic wounds.^[1]^ MU is mostly presented as well-differentiated squamous cell carcinoma (SCC) with a more aggressive nature, higher recurrence rate, and poorer survival than non-MU SCC.^[2]^ MU specific to pressure ulcers is even rarer, with an incidence as low as 0.5% to 2.6% among all MUs.^[3]^ However, these patients had higher possibilities of metastasis up to 60% than those with MU of other etiologies.^[4]^

Given its poor prognosis, early diagnosis of malignant change in pressure ulcer cannot be overemphasized. Malignant pressure ulcer degeneration is characterized by the appearance of a mass, new onset of pain, or a change in the odor, volume, or appearance of drainage.^[5]^ However, not all MUs have typical manifestations, for example, MU in spinal cord injury patients who are lack of sensation,^[6]^ or MU superimposed infection,^[7]^ and these would make the diagnosis more difficult and thus delay the treatment. Herein, we presented a patient with spinal cord injury who developed MU in his ischial pressure ulcer, which initially presented as necrotizing soft tissue infection (NSTI).

## 2. Case report

The patient was a 45-year-old man with a history of spinal meningocele and underwent surgery at age 2 years. He experienced lower-limb weakness after the surgery, and he could walk with an assistive device since his childhood. He had a history of right ischial pressure sore complicated with perineum Fournier gangrene and ischial bone osteomyelitis status post serial debridement 7 years ago. A chronic right hip dislocation was also noted, and he was lost to follow-up after recovery from infection.

**Figure 1.**
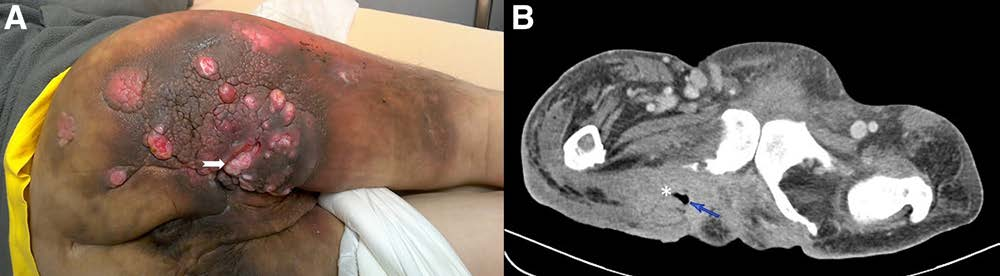
Initial presentation of a 45-year-old man with right buttock and thigh necrotizing soft tissue infection. (A) Clinical picture showing thickened skin with hyperpigmentation and multiple discharging sinuses. An ischial pressure ulcer at the center. (arrow) (B) Contrast-enhanced computed tomography image showing the soft tissue mass (star sign) with free air (arrow).

At this time, he presented with right buttock and proximal thigh erythematous swelling with pus formation that persisted for days (Fig. 1A). On admission, physical examination revealed an inflamed, hyperpigmented skin lesion with multiple discharging sinuses at the right buttock. At the center of the skin lesion, a deep ischial pressure sore that connected to the bone was noted. Laboratory examination showed leukocytosis with a predominance of neutrophils (white blood cell count, 17,590/µL; neutrophils, 87.5%). Computed tomography (CT) revealed a large soft tissue mass and fluid accumulation at the right buttock and lateral thigh with fascial thickening, gas bubble, and subcutaneous fat stranding, compatible with NSTI (Fig. 1B). Thus, he underwent emergent debridement and received empirical antibiotic therapy (ceftriaxone 2 gm *Q* 12 hours).

**Figure 2.**
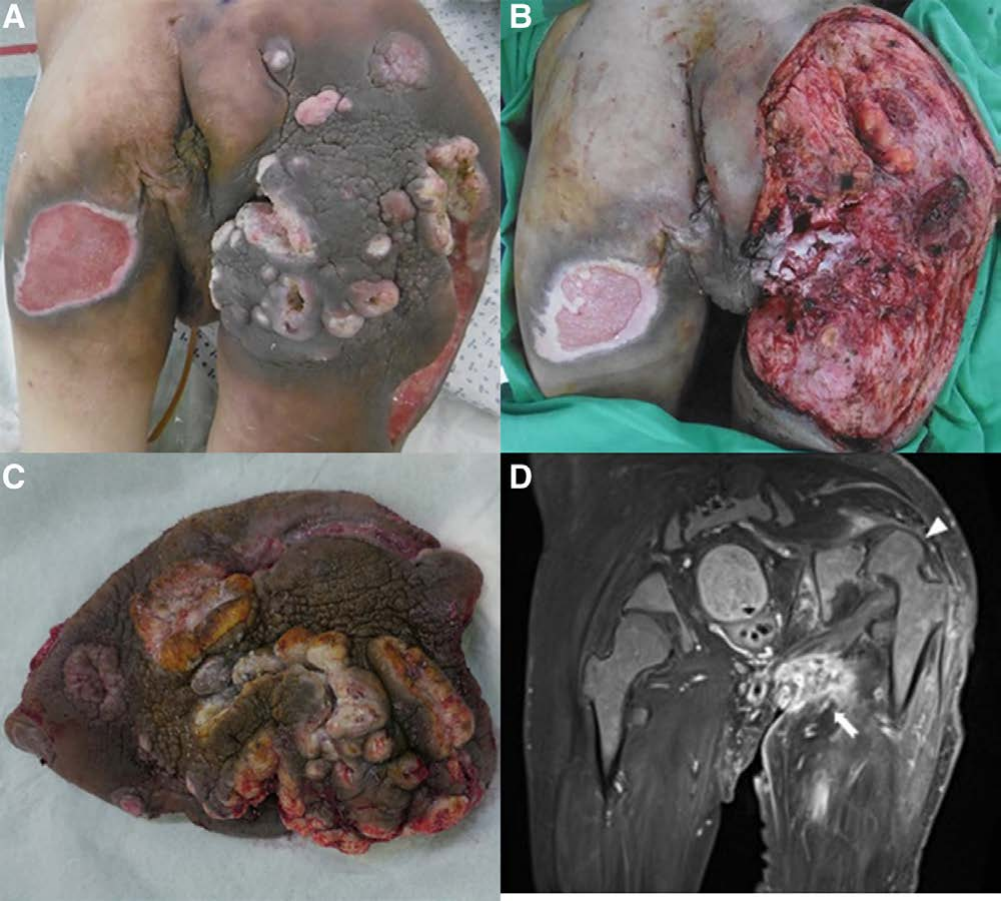
(A) Clinical picture post-serial debridement and 2-week antibiotic treatment. (B, C) Wide excision of the mass that was later confirmed as a squamous cell carcinoma. The dissection depth included a part of the gluteal muscle, and the ischium was exposed. (D) Postoperative gadolinium-enhanced T1-weighted magnetic resonance image revealed residual infiltrative ulcerative tumor involving the internal obturator and gluteus maximus muscles. (Arrow) Cephalic dislocation of the right hip joint (arrowhead).

The wound culture revealed polymicrobial infection, including *Haemophilus parainfluenzae, Staphylococcus aureus*, B-Streptococcus group G, and *Peptostreptococcus anaerobius*. The antibiotic was shifted to Flumarin 1 gm *Q* 12 hours. He then underwent 2 more sessions of debridement, and the infection subsided after 2 weeks. However, the verruca-like skin lesion remained (Fig. 2A), and previous biopsy showed squamous hyperplasia with focal severe dysplasia. With suspicion of skin malignancy and for better wound healing, wide excision and colostomy diversion surgery were performed simultaneously. However, during the excision, no obvious surgical plane was found, and tissue hypervascularity caused massive bleeding of up to 2600 cc. After the surgery, the wound was kept open for wet dressing (Fig. 2B). The excised mass was sent to pathology (Fig. 2C).

The pathology showed well-differentiated SCC with a positive margin at the buttock base. Since the diagnosis of MU was confirmed, a thorough survey was arranged, including magnetic resonance imaging (MRI), chest CT, and bone scan. MRI revealed a residual tumor in the right buttock with involvement of the gluteus maximus (Fig. 2D) and enlargement of the inguinal lymph nodes. Chest CT and bone scan did not detect definite metastasis. Because of long-term hip dislocation and dysfunctional right leg, we performed hip disarticulation with the patient’s consent.

**Figure 3.**
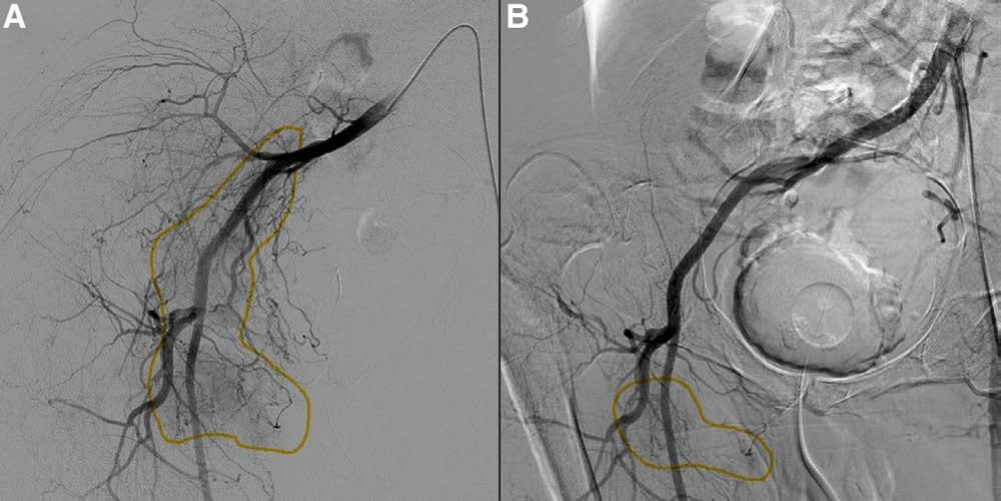
(A) Angiogram showing a hypervascular tumor stain in the right buttock and thigh (circle), mainly supplied by the superior gluteal, inferior gluteal, iliolumbar, obturator, internal pudendal, and lateral circumflex femoral arteries. (B) After embolization of the branches of the right internal iliac artery, the angiogram showed devascularization of the tumor at the right buttock and a residual stain at the right thigh (circle).

**Figure 4.**
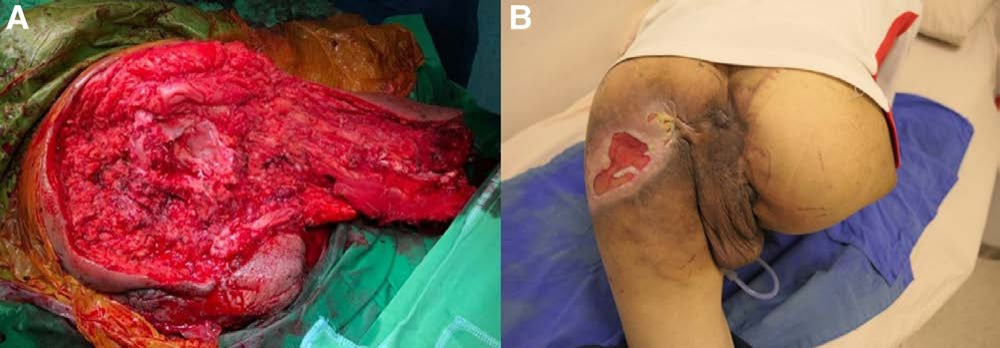
(A) Intraoperative view of hip disarticulation surgery and anterior thigh fillet flap. (B) One month after disarticulation surgery.

To reduce the blood flow of the tumor, a transarterial embolization (TAE) was arranged 1 day preoperatively. The angiogram revealed a hypervascular tumor, and the tumor stain decreased nearly 80% after TAE (Fig. 3). Right hip disarticulation with anterior thigh fillet flap was completed uneventfully with an estimated blood loss of 200 cc (Fig. 4), and lymph node dissection was not performed at the same time because of the lateral position. The final pathology revealed a 9 × 8 cm SCC with muscle invasion, but no bony, perineural, or lymphovascular invasion, and the resection margins were free of tumor. Therefore, the radiation oncologist suggested no radiotherapy required. The wound finally healed well, and the patient was discharged.

**Figure 5.**
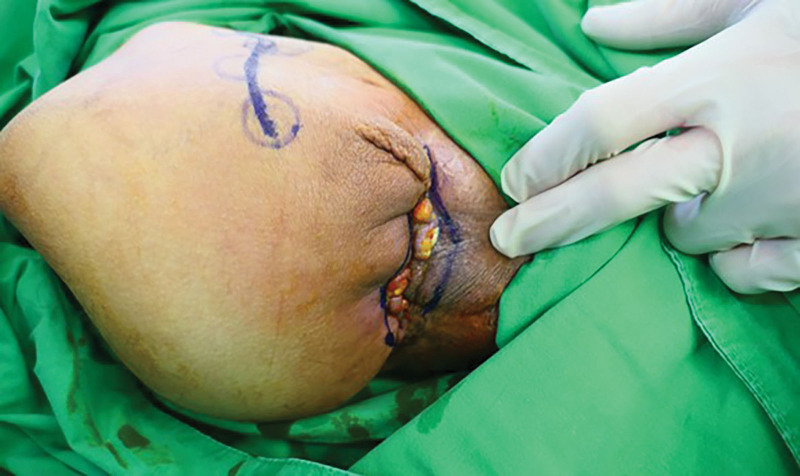
Local recurrence was noted at the wound edge between the fillet flap and perineum. Persistent inguinal lymph adenopathy was also marked.

**Figure 6.**
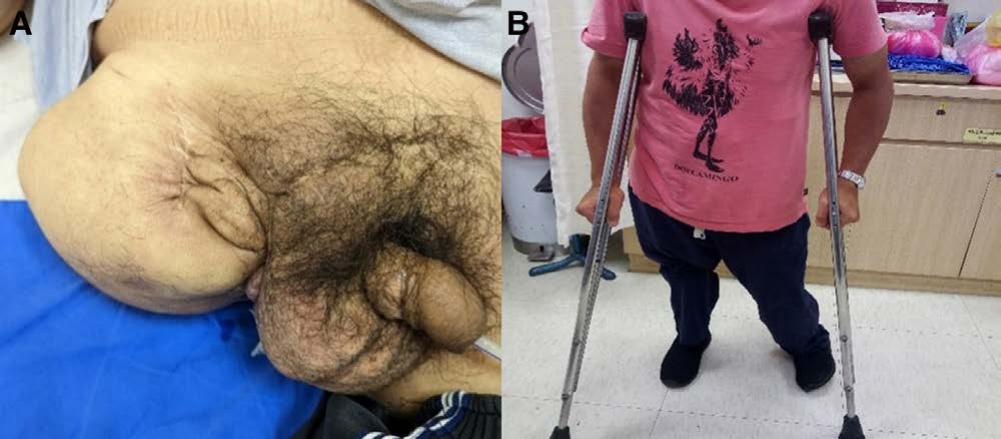
(A) The latest follow-up at 34 months post 2^nd^ wide excision surgery. (B) The patient can walk with a hip prosthesis and crutches.

However, 3 months later, a nodular lesion with ulceration developed at the previous wound edge of the perineum, and the inguinal lymph node enlargement persisted (Fig. 5). With the impression of local recurrence, he underwent wide excision and inguinal lymph node dissection. The pathological examination confirmed SCC with negative margins, and lymph node metastasis was noted (0/15). Postoperatively, he underwent adjuvant radiotherapy with a dose of 66 Gy. Thereafter, recurrence or metastases were not noted during his last follow-up visit 34 months after the second surgery. As of this writing, the patient can move with a wheelchair or a hip prosthesis and is partially dependent for daily activities. (Fig. 6)

## 3. Discussion

The incidence of MU specific to pressure ulcers is very low, but the metastasis and mortality rates are high.^[3,4]^ The aggressive clinical course of MU in pressure ulcers necessitates early detection with prompt management. The characteristics of malignant transformation of chronic ulcer should be identified, including a necrotic center with an everted edge, malodorous discharge, exophytic growth, excess bleeding, protracted course, or increase in size despite treatment.^[8]^ Although the abovementioned signs can elicit clinical suspicion, it is even difficult to differentiate MU when the case is complicated with NSTI, just like our case.

As we have known, only Takehiro Kasai et al^[7]^ reported an SCC in pressure ulcers that presented as NSTI. It was noted in a 59-year-old paraplegic patient with a stage III ischial pressure ulcer that presented as NSTI with sepsis initially and finally diagnosed as well-differentiated SCC. The initial survey detected lymph node, bone, and lung metastases, and the patient underwent palliative radiotherapy and chemotherapy and died 11 months after diagnosis. Harview et al^[9]^ also presented a case of SCC that masqueraded as necrotizing hidradenitis suppurativa at the perineal region. The patient underwent wide excision but had early local recurrence with suspected lymph node metastasis in 2 months. Neoadjuvant chemotherapy was initiated, and the final outcome was not reported in the study.

These reports and our case revealed that MU accompanied by infection is easily misdiagnosed, just like the clinical presentation of Figure 1A where to make the diagnosis of MU at this stage was difficult. Typical clinical symptoms of NSTI included soft tissue edema, erythema, severe pain, tenderness, and skin bullae or necrosis.^[10]^ While pure infective ulcers may be copious and purulent, malignant ulcers exude thick, colorless, foul-smelling fluid that dries to form a very adherent thick grayish covering.^[11]^ When the infection is under control but the ulceration fails to respond to local treatment, tissue specimens should be collected from various places of the ulcer and its margin to minimize false-negative results of histopathological examination.^[1]^ In our case, the first biopsy report was severe dysplasia, and SCC was noted after wide excision of the lesion. Therefore, a repeated or deeper biopsy was suggested in a highly suspected lesion. Additionally, because of the high recurrence rate of MU,^[3]^ repeated biopsy and close follow-up was also suggested.

Regarding the location and treatment of pressure sore-related MU, most patients with spinal cord injuries developed pressure sore-related MU in sacral and ischial areas, which were hypervascular with rich lymphatic drainage to the pelvic region, explaining the high-metastatic rate.^[12]^ It corresponded to the rapid progression toward the skeletal system, with secondary osteomyelitis,^[1,12,13]^ or even distant metastasis.^[7,14]^ Therefore, the optimal treatment for pressure sore carcinoma were early wide surgical resection and lymph node dissection; radiation was used as palliative or adjuvant treatment.^[3]^ In our case, because the initial diagnosis was only squamous dysplasia, we firstly considered wide excision with reconstruction; however, the tumor infiltrated the deep margin, and the massive intraoperative blood loss was fatal. Regarding the poor function of the right leg and the difficulty in reconstruction, hip disarticulation with a fillet flap would be a reasonable treatment choice. Preoperative TAE effectively devascularized the tumor and reduced blood loss. As a result, the surgery proceeded smoothly. Although the pathology revealed clear margin, the local recurrence within 3 months might have indicated obscure residual tumor. However, after a repeated wide excision and radiotherapy, the tumor appeared to be ablated without metastasis. Fortunately, the patient survived with satisfactory quality of life. Reviewing the treatment course of this patient, if we could have made the accurate diagnosis before wide excision via deep tissue biopsy or image study (e.g., MRI, positron emission tomography), we might have directly gone to TAE and hip disarticulation to minimize the suffering of the patient.

Compared with other studies, demonstrating > 80% mortality rate within 18 months in pressure sore-specific MU,^[15,16]^ our case showed better outcomes.^[7,12–14]^ We believe that radical excision is the key to long-term survival. Therefore, limb sacrifice is sometimes necessary, especially in SCC with bony involvement or arising from a deep pressure ulcer.^[17]^

Regarding the reconstruction method, residual cavities were large after excision of large sacral and ischial pressure sore-related MU. Therefore, the commonly used flaps for pressure ulcer reconstruction are usually ineffective in this situation. The “3 muscle flaps” using the thigh musculature and total thigh flap with lower-leg amputation have been described. ^[18,19]^ Pedicle transverse rectus abdominis myocutaneous flap,^[15]^ gluteus maximus V-Y flap,^[20]^ and Dufourmentel LLL flap had also been reported. Most straightforwardly, after limb sacrifice, a pedicle or free fillet flap can be used in hip disarticulation, hemipelviectomy, or even hemicorporectomy.^[21]^ Our case also demonstrated good wound coverage and healing process.

In conclusion, pressure sore-related MU rarely occurs, and the presentation of NSTI easily leads to misdiagnosis with focus on infection treatment per se. The presented case demonstrates the importance of awareness of the malignant potential of chronic wounds, and limb sacrifice should be considered in case of bone involvement or pressure ulcer related MU.

## Author contributions

**Conceptualization:** Ching-Yen Huang, Zhao-Yu Hsieh, Ke-Chung Chang, Dun-Hao Chang.

**Data curation:** Ching-Yen Huang, Zhao-Yu Hsieh, Dun-Hao Chang.

**Investigation:** Ching-Yen Huang, Zhao-Yu Hsieh.

**Supervision:** Ke-Chung Chang, Dun-Hao Chang.

**Writing – original draft:** Ching-Yen Huang, Zhao-Yu Hsieh.

**Writing – review & editing:** Ke-Chung Chang, Dun-Hao Chang.
